# Recurrent acute coronary syndrome and restenosis after percutaneous coronary intervention in a patient with idiopathic thrombocytopenic purpura: a case report and literature review

**DOI:** 10.1186/s12872-015-0092-3

**Published:** 2015-09-18

**Authors:** Ge Li-Sha, Chen Peng, Li Yue-Chun

**Affiliations:** Department of Pediatric, Second Affiliated Hospital of Wenzhou Medical University, Wenzhou, 325000 China; Department of Cardiology, Second Affiliated Hospital of Wenzhou Medical University, 109 Xueyuan Road, Wenzhou, Zhejiang China

**Keywords:** Acute coronary syndrome, Idiopathic thrombocytopenic purpura, Percutaneous coronary intervention, Antiplatelet therapy

## Abstract

**Background:**

Platelets play a pivotal role in the pathogenesis of acute coronary syndrome (ACS) and acute and chronic complications following percutaneous coronary intervention (PCI). Platelet inhibition is a cornerstone in the management of these patients. Idiopathic thrombocytopenic purpura (ITP) is a bleeding disorder characterized by premature platelet destruction mediated by autoantibodies. The safety of antiplatelet therapy and PCI in patients who have ACS and ITP is unknown. The aim of the present study is to discuss the management strategies for patients who have ACS and ITP and to review limited data available in the literature.

**Case presentation:**

We report the case of a patient with ITP who underwent three separate coronary interventions. The first PCI with stenting was performed in the left anterior descending artery 5 years ago while the patient suffered an anterior acute myocardial infarction, and the platelet count at admission was 90 × 10^9^/L. The patient presented with recurrent ACS and severe in-stent restenosis 5 years after the first PCI, and the platelet count at admission was 18 × 10^9^/L, and elevated to 87 × 10^9^/L after platelets transfusion. He was treated successfully with cutting balloon angioplasty under anticoagulation with unfractionated heparin and antiagregation with acetylsalicylic acid and clopidogrel. Four months later after cutting balloon angioplasty, the patient received an intracoronary stent when he once again presented with recurrent ACS in the setting of restenosis. The patient has been observed for 1.5 years without restenosis after the third PCI.

**Conclusion:**

We reviewed all the cases in the literature involving PCI and discussed the management strategies in patients with ITP and ACS. Available data suggest that PCI can be safe and feasible, and the risk–benefit equation of PCI procedures and antiplatelet therapies should be carefully evaluated, and the treatment should be individualized.

## Background

Platelets play a pivotal role in the pathogenesis of acute coronary syndrome (ACS) [1]. Treatment for ACS usually involves antiplatelet, anticoagulant, and antithrombotic therapy, and the performance of percutaneous coronary intervention (PCI) [1, 2]. In addition, platelet aggregation also plays a crucial role in the pathogenesis of acute and chronic complications following PCI [1–3]. The combination of acetylsalicylic acid and thienopyridine derivatives is a mainstay in the management of patients undergoing PCI [1–3]. All of these medications are associated with bleeding sequelae and are generally contraindicated in patients with thrombocytopenia.

Idiopathic thrombocytopenic purpura (ITP) is an autoimmune syndrome involving antibody and cell-mediated destruction of platelets and suppression of platelet production that may predispose to bleeding [4]. Spontaneous mucocutaneous bleeding is common and death from hemorrhage occurs in approximately 5 % of the ITP patients [4]. The decision to treat ITP is based on the platelet count, the degree of bleeding, and the patient’s lifestyle. Many patients with ITP require no therapy and only careful monitoring. Treatment is indicated when platelets are <30 × 10^9^/L, and is generally based on steroids and splenectomy [4]. Since the incidence of ITP is around 100 cases in 1 million persons per year, ACS patients with ITP are seen very rare. The safety of antiplatelet therapy and PCI in patients who have ACS and ITP is unknown, and to our knowledge, there are no guidelines or randomized studies to suggest treatment approaches in such patients.

We present here a case of a patient with ITP who experienced recurrent ACS and restenosis after PCI and we discuss the management strategies, including antiplatelet therapy.

## Case presentation

A 75-year-old Chinese man was admitted to our hospital with increasing chest pain and dyspnea upon exertion. The chest pain developed 4 weeks previously when he was climbing stairs, and this was relieved by several minutes rest. He also reported that 2 day preceding hospitalization, the pain was triggered by minimum exertion, appearing even at rest. His chest pain was characterized by a squeezing pattern and this was located in the substernal area and it radiated to his left arm. He had no history of hypertension, hypercholesterolemia, diabetes mellitus, smoking and drinking, and there was no history of ischemic heart disease in his family.

Eight years ago he was diagnosed of chronic ITP, but didn’t take any treatment. His platelet counts through the years had always been above 60 × 10^9^/L. This earlier diagnosis of ITP was based on the observation of moderate thrombocytopenia and antiplatelet antibodies, with bone marrow findings consistent with the diagnosis. Five years ago primary PCI was performed in the proximal left anterior descending coronary artery using a drug-eluting stent (DES) because of acute anterior ST-segment elevation myocardial infarction at other hospital. At the time of the first PCI, his platelet count had been around 90 × 10^9^/L. He received 300 mg of clopidogrel and 300 mg of aspirin along with 5000 units of heparin bolus before PCI. He was discharged on aspirin 100 mg and clopidogrel 75 mg orally daily for 1 year, and he never had bleeding before.

**Fig. 1:**
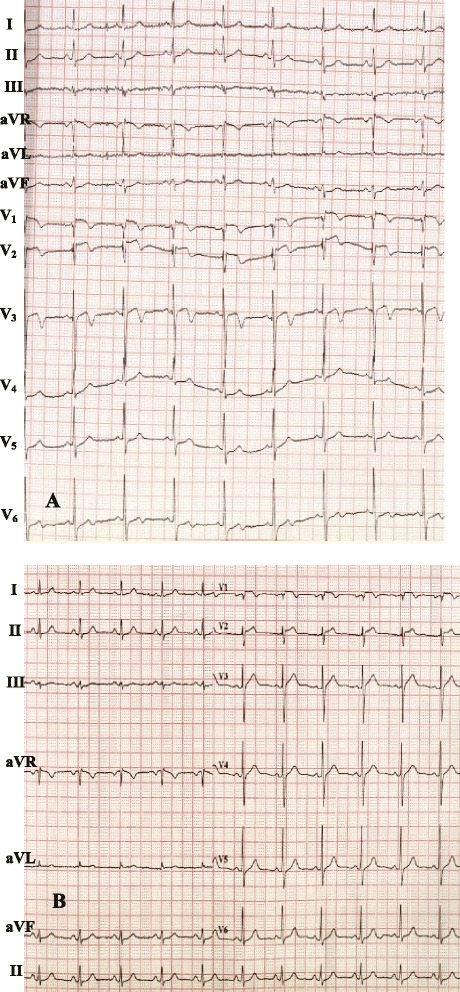
Electrocardiography on admission and after PCI. **a** Electrocardiography performed on admission showing biphasic or inverted T waves in leads V_1–3_. **b** Electrocardiography performed after PCI showing a resolution of the T wave abnormalities in V_1-3_

**Fig. 2:**
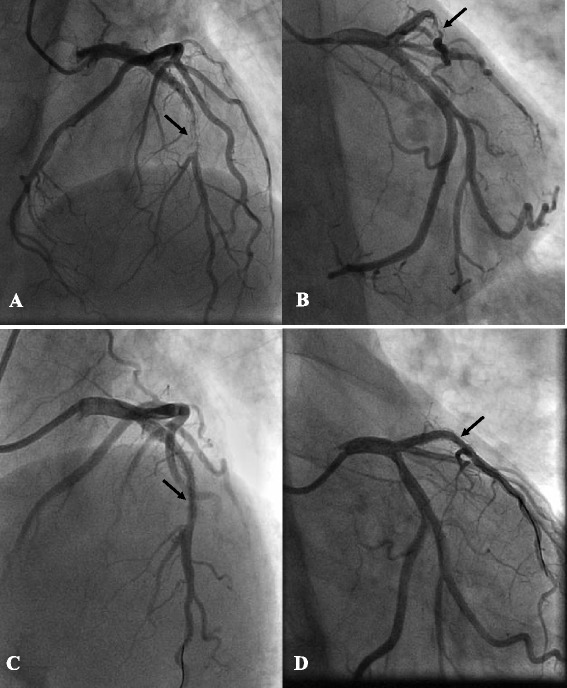
Coronary angiography in a CRA projection (**a**) and an RAO + CAU projection (**b**) showing a severe in-stent restenosis of approximately 95 % in the middle part of left anterior descending artery (arrow). Repeat left coronary angiography following successful cutting balloon angioplasty (arrow) in a CRA projection (**c**) and an RAO + CAU projection (**d**)

On physical examination, his blood pressure was 135/70 mmHg. His heart rate was regular at 76 beats/min. The chest was clear to auscultation and percussion bilaterally. There was no heart murmur. Findings of abdominal and neurological examinations were unremarkable, and lower extremities were without edema. A 12-lead ECG recorded on admission in the patient showed normal sinus rhythm and biphasic or inverted T waves in leads V_1–3_ (Fig. 1a). Blood examination revealed a platelet count of 18 × 10^9^/L, mean platelet volume 13.6 fl (normal range: 6.5–11.0), troponin I level of 0.55 ng/mL (reference, <0.1), creatine kinase of 105 IU/L (normal range: 38─171), total cholesterol of 4.47 mmol/l, low density lipoprotein-cholesterol of 1.88 mmol/l, high density lipoprotein-cholesterol of 1.40 mmol/l, triglyceride of 1.29 mmol/l and fasting blood-glucose of 5.2 mmol/l. Blood coagulation tests showed a prothrombin time of 12.4 s (normal range: 12–15), an international normalized ratio of 0.97 (normal range: 0.85–1.15) and an activated partial thromboplastin time of 35.0 s (normal: 30–45 s). Brain natriuretic peptide and chest X-ray were normal. A transthoracic echocardiography demonstrated a normal left ventricular end-diastolic internal diameter of 50 mm with a slightly decreased left ventricular ejection fraction of 0.50. Diagnosis of ACS (acute non-ST-segment elevation myocardial infarction) was made. The GRACE (Global Registry of Acute Coronary Events) risk score of the patient was 143. The patient was administered high doses of intravenous nitroglycerin and oral metrolol and atorvastatin. Ten units of platelets were transfused after the patient was assessed by a hematologist, and the platelet count elevated to 87 × 10^9^/L in the second day, and the patient was given 300 mg of aspirin and 300 mg of clopidogrel, prepared for a coronary artery angiography. Bolus injection of 3000 units unfractionated heparin was done at the beginning of the coronary angiography, and the activated clotting time (ACT) was monitored for the dose of heparin required, and then additional 3000 units were added during the PCI procedure, but GP IIb/IIIa inhibitors were not used. Coronary angiography was carried out via the radial artery, and revealed a 95 % in-stent restenosis in the middle part of left anterior descending artery (LAD; Fig. 2a, b). No other lesions were detected. The lesion in the left anterior descending artery was treated using a cutting balloon (Boston Scientific) with no complications (Fig. 2c, d). Heparin was not administered any more after the PCI, but combined anti-platelet therapy (aspirin 100 mg and clopidogrel 75 mg daily) was performed as usual. The ECG showed the T wave abnormalities in V_1-3_ were resolved after PCI (Fig. 1b). The patient was discharged asymptomatically at 3 days after PCI. Neither bleeding nor ischemic events were noted during hospitalization.

A week after discharge the platelet count was declined to 27 × 10^9^/L, and aspirin and clopidogrel were stopped in outpatient clinic. Oral methylprednisolone was begun at 1 mg/kg per day and tapered over the subsequent 6 weeks. The platelet count gradually recovered to 200 × 10^9^/L after receiving 1 week of methylprednisolone. He continued the treatment with aspirin 100 mg/day, and clopidogrel 75 mg/day for one month without any further problems.

**Fig. 3:**
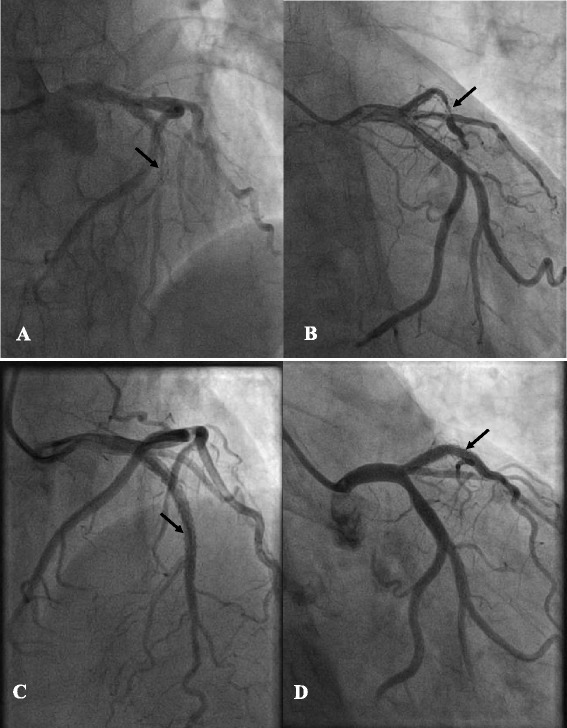
Coronary angiography in a CRA projection (**a**) and an RAO + CAU projection (**b**) showing a severe in-stent restenosis of approximately 99 % in the left anterior descending artery (arrow) 4 months after cutting balloon angioplasty. Repeat left coronary angiography after successful stenting without residual stenosis (arrow) in a CRA projection (**c**) and an RAO + CAU projection (**d**)

Four months later, the patient was readmitted due to exertional chest pain he had felt for 1 month, even though he took all the medications (aspirin, clopidogrel, metrolol, atorvastatin and oral methylprednisolone) prescribed every day. On admission, the platelet count was 124 × 10^9^/L, ECG showed normal sinus rhythm and ST segment depression in leads V_2–5_, and troponin I was mildly elevated (0.26 ng/ml; reference, <0.1). Diagnosis of unstable angina was made. Coronary angiography showed a 99 % restenosis at the site of the previous lesion and a 40 % stenosis in the proximal left circumflex artery (LCX; Fig. 3a, b). We used unfractionated heparin, a dose of 100 U/kg, aspirin 300mg and clopidogrel 600 mg were administrated. An activated clotting time of 358 s was achieved. A 3.5∗28 mm sirolimus eluted stent was deployed in the LAD with optimal angiographic result and Thrombolysis in Myocardial Infarction (TIMI) flow 3 in the LAD (Fig. 3c, d). There were no bleedings during or after the procedure, and at discharge the platelet count was 203 × 10^9^/L.

**Fig. 4:**
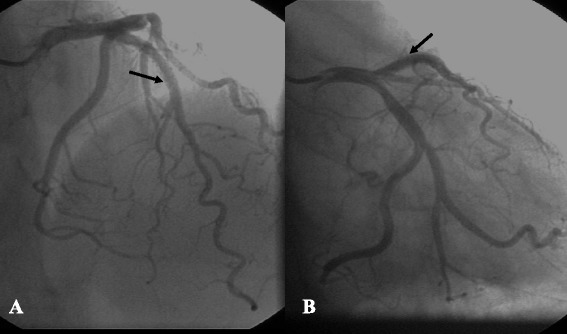
Coronary angiography in a CRA projection (**a**) and an RAO + CAU projection (**b**) showing stent patency 11 months after stenting performed in in-stent restenotic lesion (arrow)

He was then discharged on aspirin, clopidogrel, atorvastatin and oral methylprednisolone. Clopidogrel was discontinued 1 year after the procedure. During 18-month follow-up, the patient remained clinically free of symptoms without any ischemia events or bleeding complications. Coronary angiography showed stent patency at 11 months’ follow-up (Fig. 4a, b). The platelet count remained stable.

## Results in previous case reports

Previous reports focusing on ITP patients who underwent PCI were identified by searching PubMed and Google Scholar. Key words used included “ITP”, “coronary artery disease”, “ACS”, “myocardial infarction”, “angioplasty”, “PCI” and “stent”. From 1999 to January 2013, 17 studies involving 18 patients affected by ITP who underwent PCI were reported in the literature (Table 1) [5–21]. The mean age of the patients was 59.7 ± 15.1 years (range 23–80 years). Eleven patients were male, and 7 patients were female. Two patients were affected by stable angina, 4 patients by unstable angina, 6 patients by acute non-ST elevation myocardial infarction, and 6 patients by acute ST elevation myocardial infarction. Thirteen patients (72 %) had single vessel coronary artery disease (10 patients for LAD disease, 1 patient for LCX disease, 1 patient for right coronary artery (RCA) disease, 1 patient for obtuse marginal branch disease), 4 patients (22 %) had two-vessel disease, the remaining 1 patient (6 %) had three-vessel disease. The 18 patients underwent 22 PCI procedures. A total of 29 vessels [15 LAD (51.7 %), 8 LCX (27.6 %), 4 RCA (13.8 %), 1 left main coronary artery (LM; 3.4 %), 1 obtuse marginal branch of LCX (3.4 %)] were treated in the 22 PCI procedures. Re-PCI was underwent in 4 patients (22 %), 1 patient because of acute coronary thrombosis [6], 2 patients because of restenosis [10, 15], 1 patient because of staged procedure [17]. Transfemoral and transradial approaches were used for PCI in 12 and 3 patients, respectively, and approach of PCI was not specifed in remaining 3 patients. Only PTCA without subsequent stent placement were underwent in 5 of 22 procedures (22.7 %), PCI with bare metal stent placement in 13 of 22 procedures (59.1 %), and PCI with drug-eluting stent placement in 4 of 22 procedures (18.2 %). PCI was performed with extremely different platelet counts, ranging from 3 × 10^9^/L to 322 × 10^9^/L (mean 78.5 ± 81.5 × 10^9^/L). Glycoprotein IIb/IIIa inhibitors were administrated during PCI in 4 PCI procedures, clopidogrel was administrated before and during PCI in 9 PCI procedures, ticlopidine in 1 PCI procedure, acetylsalicylic acid in 9 PCI procedures, and no any antiplatelet agents were administrated before and during PCI in 5 PCI procedures. Twelve patients (66.7 %) received steroid therapy, 10 patients (55.6 %) received intravenous immunoglobulin (IVIG) therapy, and platelet transfusions were completed in 4 patients (22.2 %). One instance (5.6 %) of major bleeding (large hematom around the puncture site of the right femoral artery) [6] and 6 of minor bleeding (ecchymoses in 4 patients, small hematoma in 1 patient, diffuse petechiae in 1 patient) were observed. Ten patients (55.6 %) were discharged on double antiplatelet therapy of acetylsalicylic acid and clopidogrel, and 3 patients only on aspirin or ticlopidine, and 3 patients did not receive any antiplatelet agent at discharge. In 2 reports, antiplatelet therapy at discharge was not specifed.

**Table 1:** Case reports on percutaneous coronary intervention in patients with idiopathic thrombocytopenic purpura

Study	Pt age (Y) and sex	CAD	Pre-PCI PLT count (×10^9^/L)	Antiplatelet agent before and during PCI	Treated vessels	PCI and approach	Treatment of ITP	Bleeding	Restenosis	Discharge therapy
Fuchi et al., 1999 [20] (twice)	72, F	NSTEMI	59	None	LAD	PTCA, Femoral	STER	Large hematoma	NA	None
		STEMI	23	None	LAD	PTCA, Femoral	STER IVIG PLTT	No	NA	
Caputo et al., 2000 [6]	62, M	Unstable angina	3	NA	LAD	BMS stenting, Radial	STER	No	NA	ASA Clop
Segal et al., 2001 [21]	49, M	NSTEMI	41	ASA Clop Abciximab	RCA, LCX	BMS stenting, Femoral	STER	No	NA	ASA Clop
Kikuchi et al., 2002 [22]	68, F	STEMI	22	Ticlopidine	LAD	BMS stenting, Femoral	NA	No	No	Ticlopidine
Méndez et al., 2004 [23]	70, M	NSTEMI	170	ASA Clop Abciximab	RCA, LCX	BMS stenting, Radial	IVIG PLTT	ECC	NA	NA
Stouffer et al., 2004 [24] (twice)	77, M	Unstable angina	64	ASA	LCX	PTCA	None	No	yes	ASA
		NSTEMI	78	ASA Eptifibatide	LCX	BMS stenting	STER	Petechiae	No	ASA Clop
Amit et al., 2005 [25]	46, M	NSTEMI	38	ASA	LAD	PTCA	IVIG	No	NA	ASA
Marques et al., 2005 [26]	54, M	Unstable angina	15	None	LAD, LCX	BMS stenting, Brachial	STER IVIG PLTT	No	NA	None
Kim et al., 2006 [27]	47, F	STEMI	21	Clop	RCA	BMS stenting, Femoral	IVIG	ECC	NA	ASA Clop
Fong et al., 2006 [28]	71, F	NSTEMI	119	ASA	LAD	DES stenting, Radial	STER IVIG	No	No	ASA Clop
Park et al., 2007 [29] 3 times	61, F	Stable angina	4	None	None	CAG, Femoral	STER	Hematoma	No	None
			34	None	LAD, LCX	BMS stenting, Femoral	STER IVIG PLTT	No	yes	None
			20	None	LAD, LCX	PTCA, Femoral	STER	No	NA	None
Gracia et al., 2008 [30]	37, M	STEMI	39	ASA Clop	LAD	BMS stenting, Femoral	None	No	NA	ASA Clop
Moretti et al., 2008 [31] (twice)	66, M	Unstable angina	110	NA	RCA, LCX	BMS stenting	STER IVIG	No	NA	ASA Clop
			200	NA	LM, LAD	DES stenting, Femoral	STER	No	NA	ASA Clop
Can et al., 2009 [32]	76, M	Stable angina	100	Clop	LAD	BMS stenting, Femoral	Danazol	No	NA	NA
Yildiz et al., 2010 [33]	23, F	STEMI	35	Clop	LAD	BMS stenting, Femoral	STER	ECC	NA	ASA Clop
Neskovic et al., 2010 [34]	80, M	STEMI	5	ASA Clop	LAD	BMS stenting, Femoral	STER Danazol	No	NA	ASA Clop
Torbey et al., 2013 [35] 2 cases	61, F	STEMI	322	ASA Clop Abciximab	LAD	DES stenting, Femoral	SPL STER IVIG	ECC	NA	ASA Clop
	55, M	NSTEMI	208	Clop	Obtusemarginal branch	DES stenting, Femoral	STER IVIG	No	yes	ASA Clop

*Pt* patient, *Y* years, *F* femal, *M* male, *CAD* coronary artery disease, *NSTEMI* non-ST elevation myocardial infarction, *STEMI* ST elevation myocardial infarction, *PCI* percutaneous coronary intervention, *PLT* Platelet, *NA* data not available, *ASA* acetylsalicylic acid, *Clop* clopidogrel, *LAD* left anterior descending artery, *RCA* right coronary artery, *LCX* left circumflex artery, *LM* left main artery, *PTCA* Percutaneous transluminal coronary angioplasty, *BMS* bare metal stent, *DES* drug-eluting stent, *ITP* idiopathic thrombocytopenic purpura, *STER* steroids, *IVIG* intravenous immunoglobulin, *PLTT* Platelets transfusion, *SPL* Splenectomy, *ECC* Ecchymoses

## Discussion

Platelets play an important role in the atherosclerotic process and are intrinsically involved in the pathogenesis of ACS [1–3]. Thrombocytosis has been correlated with high incidence of ischemic heart disease, such as acute myocardial infarction [22]. Since platelets play a major role in thrombotic events, the association of ITP and ACS is rare. Nevertheless, acute myocardial infarction has been reported in even severely thrombocytopenic patients [5]. This implies that some factor other than platelet numbers alone is involved.

If a patient with chronic thrombocytopenia has coronary artery disease, the concomitance of known coronary risk factors, such as hypertension, diabetes, dyslipidemia, cigarette smoking or a family history for cardiovascular disease, should be considered. Sometimes, in patients without these coronary risk factors like the present case, it could be related with some other causes. First, platelets in patients with ITP was larger, younger, and more adhesive to the vascular surface [23]. In acute myocardial infarction or stroke patients, mean platelet volume is significantly increased despite a concomitant decrease in platelet count [24–26]. In the present case, the mean platelet volume of the patient was significantly increased. Previous studies have demonstrated that large platelets have a higher thrombotic potential [24–26]. Osuna et al [27] analysed the relationship between mean platelet volume and recurrent myocardial infarction, and found that increased mean platelet volume and platelet size contributed to reinfarction and death. Karpatkin [28] reported that the platelet aggregation velocity was directly in proportion to the platelet volume and correlated best with the megathrombocyte index. Second, elevated platelet microparticles in the ITP patients with ACS were observed [29, 30]. Microparticles are membrane vesicles released from many different cell types, including platelets. They have a potent pro-inflammatory effect, promote coagulation and affect vascular function [29, 30]. Circulating platelet microparticles and the risk of thromboembolic complications have repeatedly been demonstrated [29–31]. Therefore, platelet microparticles may be associated with coronary thrombosis in patients with ITP. Third, antigenic mimicry between platelets and endothelial cells may lead to damage of the platelets and the endothelium, caused by autoantibodies directed against platelet surface antigens, notably IIb/IIIa receptors [32, 33]. Finally, a rise of the platelet count with administration of steroids, intravenous immunoglobulin, or platelet transfusion in ITP might be “procoagulant” and might aggravate the potentially adverse effect of increased plasma viscosity leading to increased susceptibility to fatal thrombotic events, such as myocardial infarction or stroke [23, 34, 35].

The combination of ITP and coronary artery disease poses serious management problems in which a good balance between the prevention of thrombosis and hemorrhagic risk must be achieved. Indeed, ITP increases risks of bleeding in general and medications to inhibit platelet function are generally not recommended. However, antiplatelet agents should be used in patients with coronary artery disease unless contraindication exists, especially after stent implantation. This dilemma leads to difficulty in managing concomitant ITP and coronary artery disease. Performance of PCI in a patient with ITP presents a unique situation in which platelet function needs to be inhibited sufficiently to perform PCI safely but not to the extent that bleeding complications result. Our initial strategy was to treat the patient with drugs, but our patient had refractory symptoms and ischemic ECG changes and elevated troponin I despite therapy with nitrates, β-blockers and statins, and revascularization was, therefore, indicated. Since our patient was categorized as having high risk for major bleeding and had only a single coronary lesion, PCI was a better choice than bypass surgery. We decided to administer antiplatelet agents after platelets transfusion, and then proceed using cutting balloon angioplasty without stent implantation because of the risk of subacute stent thrombosis in a patient in whom it was unknown whether he would be tolerant of combination therapy of acetylsalicylic acid and clopidogrel after stent implantation. The patient was given steroids following cutting balloon angioplasty, and the platelet count increased and remained normal level. The patient did receive an intracoronary stent 4 months later when he once again presented with recurrent ACS in the setting of restenosis. Coronary angiography showed stent patency 11 months after stent implantation.

Based on the results of the previous case reports [5–21, 31, 36] and present report, we concluded that PCI can be a useful strategy in patients with ITP and severe coronary artery disease even when the patient has severe thrombocytopenia, and major bleeding was rare. Both transfemoral and transradial approaches were successfully used for PCI. Implanting a bare metal stent (BMS) appears to be reasonable choice during PCI, since dual antiplatelet therapy with acetylsalicylic acid and clopidogrel could be administered for a shorter period of time to allow stent endothelialization. DES implantation was also reported in 4 patients, and 5 cases of PCI without subsequent stent placement were also reported. Perioperative ITP treatment certainly minimized the complication rate in the PCI population as well. Steroids should be first choice to increase the platelet count, but if a rapid platelet count elevation were required, platelet transfusions and IVIG supplementation could be administered. A safe cutoff platelet count above which invasive procedures can be performed has not been yet established. Russo et al. [36] have suggested that when the platelet counts >50 × 10^9^/L intervention either percutaneously or surgically can be safely performed. The combined therapy of acetylsalicylic acid and clopidogrel after PCI was well tolerated in most of patients. In addtion, most of patients had single vessel coronary artery disease and LAD artery was the most commonly occluded of the coronary arteries in patient with ITP, which was the same with the general population. Among 7 cases who underwent reexamined coronary angiography, 3 patients had coronary restenosis. The restenosis developed after percutaneous transluminal coronary angioplasty without stent placement in 1 patient, after BMS implant in 1 patient, and after DES implant in 1 patient. The present case also occurred repeatedly in-stent restenosis. Repeat revascularization was successfully done in all 4 patients. Many factors may be involved in the pathophysiology of restenosis. Norgaz et al [37] found that mean platele volume before PCI is correlated with subsequent development of in-stent restenosis, and if preprocedural mean platele volume is greater than 8.4 fl, restenosis is more probable to occur. Generally, The mean platelet volume in patients with ITP was significantly increased. Therefore, platelet size may play a role in the development of restenosis after PCI in a patient with ITP.

## Conclusions

Available data suggest that PCI can be safe and feasible, and the risk–benefit equation of PCI procedures and antiplatelet therapies should be carefully evaluated, and the treatment should be individualized.

## Consent statement

Written informed consent was obtained from the patient for publication of this case report and any accompanying images.

## Abbreviations

*ACS*: Acute coronary syndrome
ACT: Activated clotting time
BMS: Bare metal stent
DES: Drug-eluting stent
GRACE: Global Registry of Acute Coronary Events
ITP: Idiopathic thrombocytopenic purpura
IVIG: Intravenous immunoglobulin
LAD: Left anterior descending artery
LCX: Left circumflex artery
PCI: Percutaneous coronary intervention
RCA: Right coronary artery
TIMI: Thrombolysis in Myocardial Infarction
